# Hyperreactio luteinalis incidentally found in the second trimester of pregnancy with normal hCG levels: a case report

**DOI:** 10.11604/pamj.2021.39.75.29745

**Published:** 2021-05-26

**Authors:** Sawssan Ben Halima, Hana Hakim, Sahbi Kebaili, Nadia Ben Jdidia, Khaled Trigui, Kais Chaabane

**Affiliations:** 1 University of Medicine of Sfax, Department of Gynecology and Obstetrics, Hedi Chaker Hospital, 3029 Sfax, Sfax, Tunisia

**Keywords:** Hyperreactio, luteinalis, ovaries, pregnancy, case report

## Abstract

Hyperreactio luteinalis (HL) is a rare entity in which both ovaries are multicystic and enlarged under the action of human chorionic gonadotropin (hCG), mostly seen in the third trimester of pregnancy. This benign condition is usually asymptomatic and doesn’t need any specific treatment, as the ovaries spontaneously reduce in size after birth. This is a case report of a 33-year-old woman diagnosed with hyperreactio luteinalis during the second trimester of her induced pregnancy. An ultrasound scan at 22 weeks of gestation revealed bilateral multicystic enlarged ovaries along with multiple fetal malformations and hydropsfetalis. Usually, HL is most commonly seen in situations in which there are high levels of hCG, but our patient had normal levels of hCG during all her pregnancy, which makes our case even rarer. In conclusion, the most important challenge when faced with HL is to differentiate between it and other differential diagnosis especially malignant tumors, because unlike them, this benign condition doesn’t need surgical treatment.

## Introduction

Hyperreactio luteinalis (HL) is a rare benign condition characterized by bilateral ovarian cystic enlargement [1]. HL is a pregnancy-related entity and is caused by elevated levels of human chorionic gonadotropin (hCG) or by an exaggerated ovarian response to this hormone [2]. Due to its rareness, HL is often mistaken for other entities, especially ovarian hyperstimulation syndrome (OHSS) and malignant tumors, which may lead to unnecessary surgical interventions. We report here a rare case of hyperreactio luteinalis in a 22-week pregnant woman diagnosed in the second trimester of pregnancy with normal hCG levels, and we discuss the particularities of this condition mainly means of diagnosis and management.

## Patient and observation

This was a 33-year-old G1P0 woman who was presented to our emergency unit for preterm premature rupture of membranes (PPROM) at 22 weeks of gestation. This patient had a 3-year history of infertility prior to this pregnancy, and was diagnosed with Polycystic Ovarian Syndrome (PCOS) and endometriosis. Two years ago, she underwent a laparoscopic surgery to remove an ovarian teratoma. This pregnancy was obtained with fertilized in-vitro embryo transfer.

**Figure 1:**
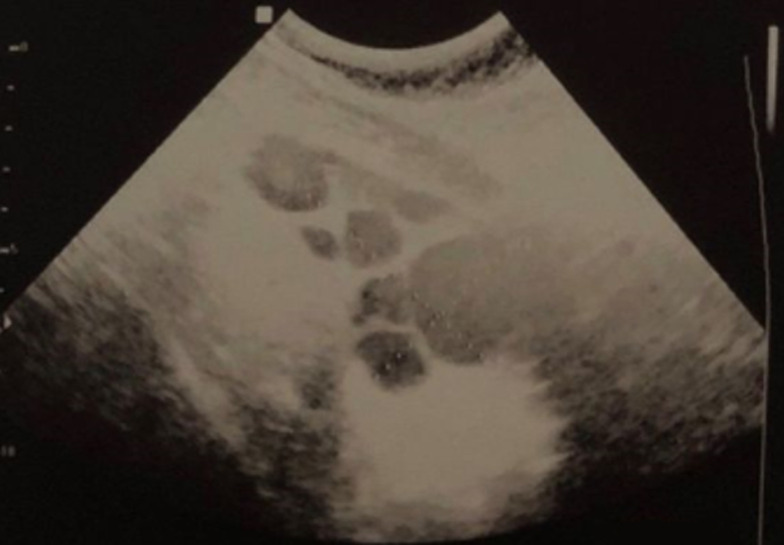
right ovary measuring 109 x 70 mm with multiple thin-walled cysts

First trimester checkups revealed no anomalies. hCG levels at 6 weeks were normal at 8,452 mIU/mL. A first ultrasound scan was performed at 6 weeks to confirm the site of the pregnancy, and it revealed an intrauterine gestational sac containing a viable embryo with demonstrable fetal cardiac activity. Both ovaries were of normal size, and a 34x30 mm corpus luteum was identified in the right ovary. There was no ascites. The patient didn´t report any abdominal cramps or dyspnea, only minor nausea and vomiting especially in the morning. A second ultrasound scan was performed at 12 weeks. The nuchal translucency was normal. Ovaries were still of normal size (inferior to 45 mm). When the patient was admitted at 22 weeks of gestation after the PPROM, a morphological ultrasound scan was performed. It revealed multiple anomalies: polyhydramnios, omphalocele containing a part of the liver and the gallbladder with no intestines covered by a 35 mm membrane, short femur length and hydropsfetalis. Detailed examination of the fetal heart revealed a 3 mm infundibular ventricular septal defect with a bidirectional shunt. In addition, the ultrasound revealed giant polycystic ovaries: the right ovary measured 109 x 70mm (Figure 1) and the left ovary measured 98 x 61mm. The biggest follicle was found in the right ovary and measured 50 mm. There was no ascites, and estrogen levels were normal. For further diagnosis, an amniocentesis was performed the next day in order to determine a fetal karyotype and detect a possible genetic condition. The fetal karyotype was normal (46, XY).

**Figure 2:**
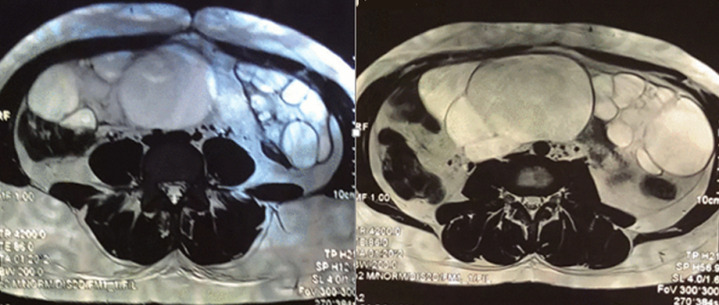
T2-weighted MRI image, axial plane through pelvis showing bilateral polycystic ovaries

However, the patient spontaneously aborted a dead fetus the next day. The anatomopathological examination of the placenta revealed no signs of amnionitis or malignancy. One-week post-abortum, an ultrasound check-up was performed, and it showed that the ovaries were still huge. Based on the ultrasonographic aspect of the ovaries, hyperreactio luteinalis (HL) was the most likely diagnosis, but malignancy couldn´t be ruled out. Tumor markers ACE and CA 125 were performed, and were in the normal range. A magnetic resonance imaging (MRI) was performed the next day: both ovaries were enlarged and polycystic. The biggest follicle was found in the right ovary and measured 90mm. The right ovary measured 150 x 65 x 191 mm and the left ovary measured 110 x 61 x 122 mm. There was no ascites (Figure 2).

Upon further interrogation, the patient revealed that atypical virilization symptoms appeared at the start of the fourth month of pregnancy, such as hirsutism (on the face, lower abdomen and thighs) and a deeper voice. Laboratory tests were ordered to further clarify the diagnosis. The results showed elevated levels of total testosterone at 16.15 ng/ml (reference range is 0.1 to 0.9 ng/ml), Delta 4 Androstenedione at 8.60 ng/ml (reference range is 0.40 to 2.80 ng/ml) and 17-hydroxyprogesterone (17-OHP) at 12.5 ng/ml (reference range is 1.0 to 4.0 ng/ml). Additional studies including thyroid-stimulating hormone (TSH), free thyroxine, dehydroepiandrosterone-sulfate (DHEA-S), cortisol and adrenocorticotropic hormone were all within normal limits. These results further supported the diagnosis of HL.

We opted to keep tracking the evolution of the size of the ovaries by serial USG scans. We noticed that both ovaries reduced in size to 104 x 91 mm for the right ovary and 76 x 63 mm for the left ovary two months after the abortion. Five months after the abortion, both ovaries were back to a normal size: right ovary at 41 x 27 mm and left ovary at 34 x 28 mm. Total testosterone levels were also back to normal levels at 0.47 ng/ml, and we noted that the virilization symptoms considerably decreased. The patient was satisfied with our management of her case especially that we made the right choice to not operate.

## Discussion

Hyperreactio luteinalis is a rare condition resulting in a benign enlargement of the ovaries during pregnancy under the influence of hCG [1]. HL is often encountered in situations where abnormally elevated hCG levels are detected, such as multiple pregnancies, hydropsfetalis, and especially gestational trophoblastic diseases: HL is encountered in 10% of cases of choriocarcinoma and in 25% of cases of molar pregnancy [3]. Less frequently, this condition can also develop in patients with increased ovarian stroma sensitivity to hCG such as PCOS [2], or in cases of diabetes mellitus and ovulation induction [1]. In addition, there is a similarity between the structure of thyroid-stimulating hormone (TSH) and hCG, which may lead to HL in women with thyroid affections [4]. In our case, hCG levels were normal, but our patient still had three of theabove-mentioned risk factors: PCOS, ovulation induction and hydropsfetalis.

Patients with HL are generally asymptomatic, but they can develop minor signs like abdominal pain (in 52% of cases), nausea, ascites and dyspnea [5]. Moreover, physicians should always keep in mind that these large ovaries carry the risk of torsion or rupture, which can affect the patient´s ulterior fertility and at most lead to hemorrhagic shock. Virilization signs (hirsutism, acne, alopecia, voice deepening) are also a common occurrence in HL, reported in 25% of cases [6]. This occurrence is explained by increased androgen levels, and is caused by PCOS in 82% of cases [7]. Our patient did not suffer from abdominal pain or nausea, but only reported a deepening voice and hirsutism at the start of the fourth month of pregnancy, along with elevated testosterone, Delta 4 Androstenedione and 17-OHP levels.

In the past, HL was usually fortuitously discovered at the time of the caesarean section. Nowadays, however, HL is usually diagnosed during the pregnancy upon visualization of multiple large theca-lutein cystsin both ovaries during one of the mandatory ultrasound exams, realizing the “spoke wheel sign”, or the appearance of a “bunch of grapes” [4]. On MRI, the ovaries appear as giant cystic masses with homogeneous low intensity on T1-weighted imaging and high intensity on T2-weighted imaging [8].

HL is often mistaken for malignant tumors or ovarian hyperstimulation syndrome (OHSS). To distinguish HL from malignant tumors such as mucinous borderline tumors, tumor markers should be tested; they are negative in the case of HL. Ultrasound and MRI are also necessary to rule out malignancy. Differentiating between the two entities is critical because women may undergo unnecessary surgical interventions during their pregnancy when a malignant tumor is strongly suspected. OHSS is even more similar to HL. However, OHSS occurs in the luteal phase or early first trimester of pregnancy whereas HL is usually encountered in the second or third trimester [5,9]. HL is typically associated to virilization signs, but these signs are a very rare occurrence in OHSS [5]. Finally, HL usually is asymptomatic or causes mild symptoms whereas OHSS usually causes abdominal pain and acute fluid imbalance that can lead to severe dyspnea, ascites, hemoconcentration, electrolyte disturbances, kidney failure and thromboembolic events [9].

Management of HL during pregnancy is ideally conservative [1,6]. Both ovaries typically get back to a regular size four to six months after delivery or abortion [6]. Surgical treatment should be reserved for complications such as ovarian torsion or cyst rupture that may even lead to hemorrhagic shock.

## Conclusion

Hyperreactio luteinalis is a rare pregnancy-related condition that causes enlargement of the ovaries. Ultrasound is the key to diagnosis, along with MRI in case of doubt. It is crucial to differentiate between HL and other differential diagnosis especially malignant tumors, because unlike them, this benign condition doesn´t need surgical treatment unless there is a complication, like torsion or rupture.
